# Assessment of the validity of *Saurida microlepis* (Aulopiformes, Synodontidae): An integrative morphological and genetic analysis

**DOI:** 10.3897/zookeys.1272.175090

**Published:** 2026-03-06

**Authors:** Hui Liu, Yufei Wang, Xiaojing Song, Chunyan Ma, Hanye Zhang

**Affiliations:** 1 East China Sea Fisheries Research Institute, Chinese Academy of Fishery Sciences, Shanghai, China Shanghai Ocean University Shanghai China; 2 College of Marine Living Resource Sciences and Management, Shanghai Ocean University, Shanghai, China Chinese Academy of Fishery Sciences Shanghai China; 3 School of Life Sciences, East China Normal University, Shanghai, China East China Normal University Shanghai China; 4 Key Laboratory of Fisheries Remote Sensing, Ministry of Agriculture and Rural Affairs, Shanghai, China Ministry of Agriculture and Rural Affairs Shanghai China

**Keywords:** China, COI gene, integrative taxonomy, lizardfish, morphology, *
Saurida
*, species validity

## Abstract

The validity of *Saurida microlepis* has long been debated, owing to close morphological similarity with congeners and the unavailability of the type specimen. We re-evaluated the species validity of *S. microlepis* through integrative analyses of morphological traits and mitochondrial cytochrome *c* oxidase subunit I sequences from 108 specimens collected along the coastal waters of China, including the type locality. Specimens were divided into three groups according to vertebral, lateral-line, and pre-dorsal-fin scales: forms matching *S. eso*, previously included as a synonym of *S. elongata*; forms corresponding to *S. microlepis*; and forms showing broad ranges across these traits. Morphological analyses revealed extensive overlap in both meristic and morphometric characters, demonstrating that they are insufficient for reliable discrimination. Genetic analyses revealed low intraspecific divergence and no distinct phylogenetic clustering. The results do not support recognition of *S. microlepis* as a valid species, and it is here confirmed as a junior synonym of *S. eso*. The different distribution patterns of *S. eso* and *S. microlepis*, combined with divergences in vertebral, lateral-line, and pre-dorsal-fin scales, suggest they may represent distinct populations of the same species. This study demonstrates the importance of combining morphological and molecular evidence to resolve taxonomic challenges in marine fishes.

## Introduction

The genus *Saurida* Valenciennes in Cuvier & Valenciennes, 1850 (Aulopiformes: Synodontidae) is predominantly distributed throughout the Indo-West Pacific (Inoue and Nakabo 2006; Bogorodsky et al. 2014). Persistent debate remains regarding the taxonomy and species delimitation within *Saurida*, largely due to the high degree of external morphological similarity (Inoue and Nakabo 2006; Russell et al. 2015; Furuhashi et al. 2023; Santanumurti et al. 2024).

*Saurida microlepis* Wu & Wang, 1931 was described based on specimens collected from Chefoo, Yantai, China. Diagnostic characters were reported, including 24 pre-dorsal-fin scales and 71 lateral-line scales. The latter was regarded as a key distinguishing character relative to all other known species in China, Japan, and the Indo-Australian Archipelago. The holotype of *S. microlepis* was deposited at the Museum of Biological Laboratory of Science Society of China in Nanjing, but is considered to have been destroyed during wartime (Russell et al. 2024). Despite a relatively detailed description by Wu and Wang (1931), the taxonomic validity of *S. microlepis* has remained uncertain. Norman (1935) was the first to comprehensively revise the genus *Saurida*, suggesting that the increased lateral-line scales reported by Wu and Wang (1931) included an additional 4–5 scales at the caudal-fin base. When this character was excluded, Norman (1935) considered *S. microlepis* morphologically indistinguishable from *S. elongata* (Temminck and Schlegel 1846). The designation of *S. microlepis* by Norman (1935) as a queried junior synonym of *S. elongata*, was subsequently supported by Matsubara and Iwai (1951) and Chen (2002), although later studies (Yamada et al. 2007; Nakabo 2013; Yeo and Kim 2018; Wu and Zhong 2021) identified morphological differences in lateral-line scales, vertebral numbers, and pre-dorsal-fin scale counts that support the recognition of *S. microlepis* as a valid species. However, Russell et al. (2024) recently pointed out that *S. elongata* has been much confused, and the name has long been misapplied. Re-examination of the three type specimens of *Aulopus elongatus* Temminck & Schlegel, 1846 in the Naturalis Biodiversity Centre, Leiden, by Russell et al. (2024) showed that the types of *A. elongatus* comprise two distinct species, with two of the specimens (including the lectotype) of *S. elongatus* being indistinguishable from *S. wanieso* Shindo & Yamada, 1972, which they relegated as a junior synonym of *S. elongatus*; the third specimen closely matching *S. eso* (Jordan & Herre, 1907), which they resurrected as a valid species. They also included *S. microlepis* as a junior synonym of *S. eso*, although with only limited justification.

**Figure 1.**
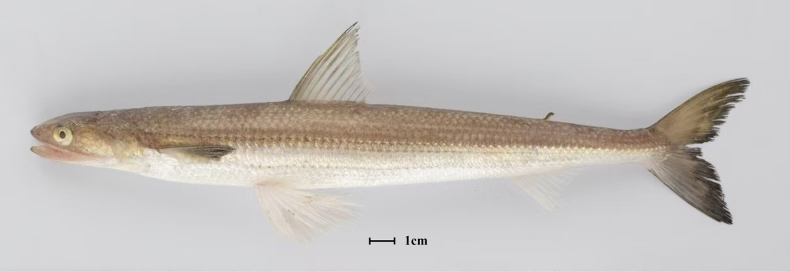
A specimen (ZYSSZ053) collected from the type locality (Chefoo, Yantai) of *Saurida microlepis*. Photo by Hanye Zhang. Scale bar: 1 cm.

Because of the absence of the holotype of *S. microlepis* and the inadequacy of key diagnostic features in the original description, the taxonomic status of *Saurida microlepis* has remained unresolved. *Saurida microlepis* and *S. eso* have short pectoral fins that do not reach the origin of the pelvic fins. This diagnostic feature distinguishes them from all other *Saurida* species in coastal waters of China, except for *S. micropectoralis* Shindo & Yamada, 1972. However, the presence of grey blotches along the lateral body serves as the primary diagnostic morphological feature of *S. micropectoralis* (Shindo and Yamada 1972; Chen 2002; Wu and Zhong 2021). This study conducts an integrative morphological and molecular analysis of *Saurida* specimens collected from multiple coastal sites in China. All these specimens are characterized by pectoral fins that do not reach the pelvic-fin origin and the absence of lateral body blotches (Fig. 1), with the aim of clarifying the taxonomic status of *S. microlepis*.

## Material and methods

### Sample collection

**Figure 2.**
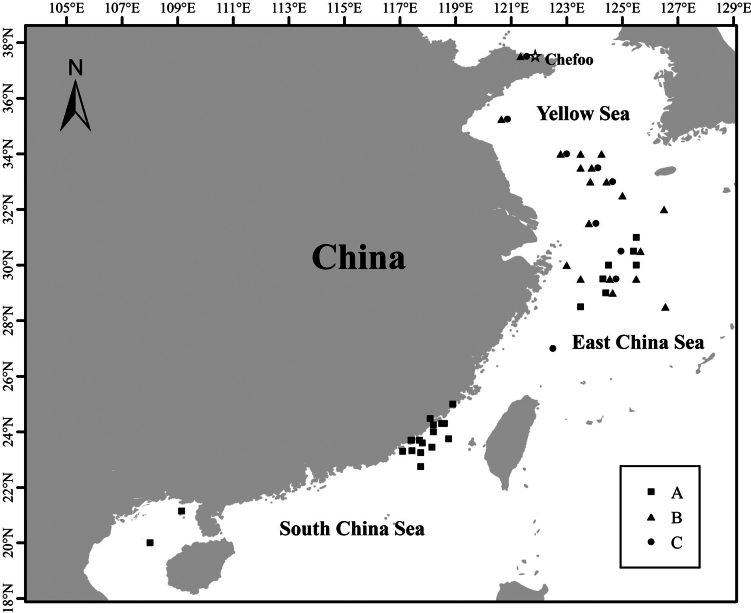
Sampling map of *Saurida* specimens from the Chinese coast. Squares, triangles, and circles indicate the sampling locations of specimens for groups A, B and C, while the star denotes the type locality of *S. microlepis*.

Samples were obtained between 2021 and 2025 from coastal waters of China, including the type locality of *Saurida microlepis*, Chefoo, Yantai (Fig. 2). Specimens were sourced from fisheries resource surveys, commercial fishing, and local markets. Specimens (voucher ZYSSZ025~ZYSSZ122) examined in this study are stored in the East China Sea Fisheries Research Institute (**ECSFRI**), Chinese Academy of Fishery Sciences (**CAFS**), Shanghai, China, and were fixed in 10% formalin.

### Specimen grouping

Based on the combination of vertebral, lateral-line scales, and pre-dorsal-fin scales counts, specimens were assigned to three groups. Group A (*N* = 40) comprised individuals with ≤61 vertebrae, ≤64 lateral-line scales, and 18–24 pre-dorsal-fin scales, matching the *S. elongata* morphological form. Group B (*N* = 55) comprised individuals with ≥61 vertebrae, ≥64 lateral-line scales, and 24–27 pre-dorsal-fin scales, corresponding to the *S. microlepis* morphological form. Thirteen specimens exhibited broad ranges across all three traits, with vertebrae 60–63, lateral-line scales 62–67, and pre-dorsal-fin scales 22–27; consequently, they could not be unambiguously classified and were designated as Group C (*N* = 13). Fig. 2 illustrates the sampling locations for groups A, B, and C. The specimens ranged widely in size, with body length and body weight ranges summarized in Table 1.

**Table 1.** Body length and body weight ranges for specimens of groups A, B, and C.

Group	Number	Body length/mm		Body weight/g	
		Range	Mean ± SD	Range	Mean ± SD
A	40	120.7–410.0	228.3 ± 62.6	11.1–601.2	148.3 ± 137.3
B	55	165.0–372.6	252.1 ± 56.1	37.2–526.8	177.6 ± 122.7
C	13	162.0–350.0	229.4 ± 51.2	39.8–391.5	138.6 ± 99.6

### Morphological analysis

#### Meristic and morphometric data measurement

**Figure 3.**
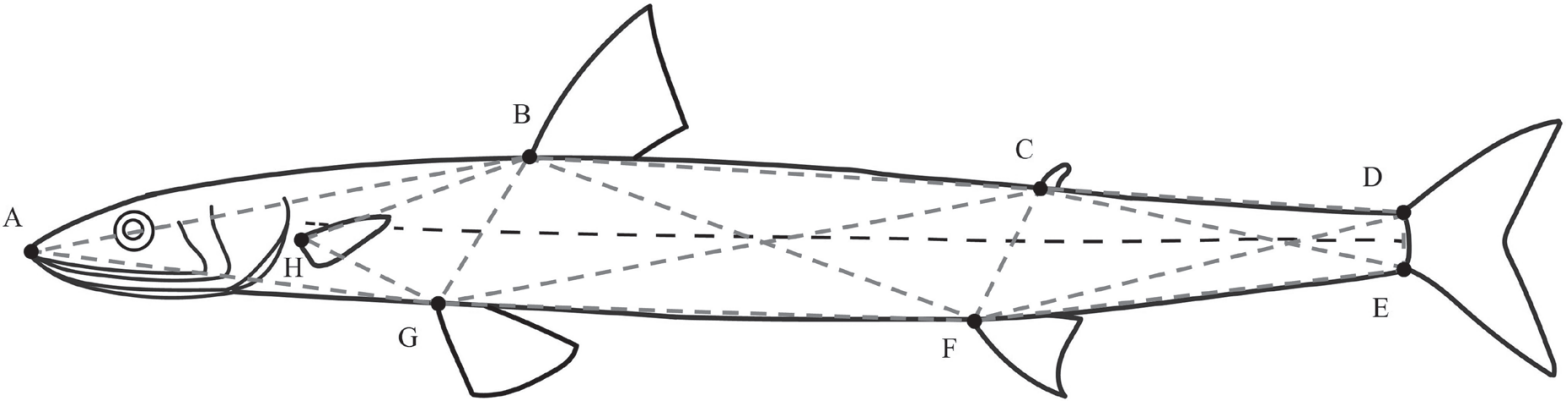
Morphological landmarks and truss network of specimens. Landmarks are defined as: **A**. Snout tip **B**. Dorsal-fin origin; **C**. Adipose-fin origin; **D**. Upper caudal-fin origin; **E**. Lower caudal-fin origin; **F**. Anal-fin origin; **G**. Pelvic-fin origin; **H**. Pectoral-fin origin.

A total of 38 morphological characters were measured, comprising 10 meristic, 13 traditional morphometric, and 15 truss dimensions. Meristic characters included counts of pectoral, pelvic, dorsal, anal, and caudal-fin rays; lateral-line scales, scale rows above and below the lateral line, and pre-dorsal-fin scales; and vertebrae, with vertebrae counted from X-ray images. Morphometric characters included body length (BL), total length (WL), head length (HL), postorbital length (EL), snout length (SL), body depth (BH), head depth (HH), pre-pectoral-fin length (AL), pre-dorsal-fin length (CL), pre-adipose-fin length (DL), pre-anal-fin length (FL), pre-pelvic-fin length (GL), and pectoral-fin length (PL). Counts and measurements followed Furuhashi et al. (2023) and Russell et al. (2024). The truss-network analysis was based on eight landmarks on the body (Fig. 3), from which 15 distances between landmarks were measured. Prior to statistical analyses, all morphometric characters were standardized to body length (BL) to remove the effect of body size variation.

### Statistical analysis

One-way analysis of variance (ANOVA) was performed on the standardized morphometric data. Principal component analysis (PCA) was then used to extract the major morphological variation among samples, with the suitability of the data confirmed by Bartlett’s test (p < 0.01). PCA analyses were performed using Origin 2022. Coefficients of difference (CD) among the three sample groups were then calculated following Mayr et al. (1953), with CD > 1.28 taken as the threshold for differentiation at or above the subspecies level.

### DNA analysis

#### DNA extraction, PCR amplification, and sequence analysis

Dorsal muscle tissue was cut from each specimen and preserved in 95% ethanol at –20 °C until DNA extraction. Genomic DNA was extracted from muscle using TIANamp Marine Animals DNA Kit (TIANGEN, China). The polymerase chain reaction was used to amplify fragments of cytochrome *c* oxidase subunit I (COI) (Ward et al. 2005).

PCR was carried out in a 25 µL reaction volume containing 9.5 µL of ddH_2_O, 12.5 µL of PCR mix (Wonbio., Ltd) containing Taq DNA Polymerase (with pre-mixed MgCl_2_, dNTP mix and buffer), 1 µL of DNA template, and 1 µL of each primer. The thermal cycling consisted of an initial denaturation at 94 °C for 4 min 30 s, followed by 35 cycles of denaturation at 94 °C for 40 s, annealing at 52 °C for 40 s, and extension at 72 °C for 40s. A final extension was performed at 72 °C for 5 min.

DNA sequences were aligned using CLUSTALW in MEGA 12 (Kumar et al. 2024). Ambiguous bases at both ends were trimmed, and all sequences were adjusted to the same length. Haplotype analyses for the sequences were conducted using DnaSP v. 5.1 (Librado and Rozas 2009).

#### Genetic diversity and phylogenetic tree construction

Based on the Kimura 2-parameter (K2P) model, pairwise genetic distances among all individual sequences were estimated by correcting for transition and transversion substitution rates. A maximum-likelihood (ML) phylogenetic tree was constructed with 1000 bootstrap replicates. The tree was based on all haplotypes, using *Saurida macrolepis* (GenBank accession number: MG574445) (Bingpeng et al. 2018) and *S. fortis* (GenBank accession number: LC881972) (Furuhashi et al. 2025) as the outgroup. All analyses were conducted in MEGA 12 (Kumar et al. 2024).

## Results

### Morphological analysis

The frequency distributions of vertebrae, lateral-line scales, and pre-dorsal-fin scales are shown in Table 2. Table 3 shows the results of the proportional measurements and counts for 108 analyzed specimens. The three groups showed no clear separation in meristic characters including vertebral, lateral-line, and pre-dorsal-fin scales. Vertebral ranged from 57 to 66, with a concentration around 61. Lateral-line scales ranged from 57 to 70, with substantial overlap around 64. Pre-dorsal-fin scales ranged from 18 to 27, with 24 occurring in all groups, indicating overlap and a continuous distribution. Other meristic characters, such as fin-ray counts and scale rows above and below the lateral line, showed substantial overlap among the three groups, similar to previous studies (Matsubara and Iwai 1951; Shindo and Yamada 1972; Chen 2002; Yeo and Kim 2018). Group A exhibited a lower minimum in pre-dorsal-fin scale count than values reported in previous studies, possibly due to scale loss or ambiguity in a few specimens. Even with this deviation, the extensive overlap in meristic traits was insufficient for reliable diagnosis.

**Table 2.** Frequency distributions of lateral-line scales, vertebrae, and pre-dorsal-fin scales for groups A (*N =* 40), B (*N =* 55), and C (*N* = 13).

Lateral-line scales														
	57	58	59	60	61	62	63	64	65	66	67	68	69	70
A	1	1	3	4	9	8	8	6						
B								19	11	4	9	8	3	1
C						4	6		2		1			
Vertebral														
	56	57	58	59	60	61	62	63	64	65	66			
A	1	1	4	13	14	7								
B							25	19	10		1			
C					1	3	6	3						
Pre-dorsal-fin-scales														
	19	20	21	22	23	24	25	26	27					
A	1	5	3	16	9	6								
B						17	21	12	5					
C	2		2	3	4			1	1					

**Table 3.** Morphological comparison of *Saurida eso* examined in this study with conspecific specimens from previous studies. Species previously recorded as *S. elongata* are essentially *S. eso*.

	This study			Jordan and Herre (1907)	Matsubara and Iwai (1951)	Shindo and Yamada		Chen (2002)	Yamada et al. (1986)		Yeo and Kim (2018)		Russell et al. (2024)
	A (*N =* 40)	B (*N =* 55)	C (*N* = 13)	*S. eso*	*S. elongata* (*N =* 114)	*S. elongata* (S) (*N* = 78)	*S. elongata* (N) (*N =* 53)	*S. elongata* (*N =* 12)	*S. elongata* (S)	*S. elongata* (N)	*S. elongata* (*N =* 2)	*S. microlepis* (*N =* 46)	*S. eso* (*N =* 36)
Counts													
Pectoral-fin rays	13–16 (14)	12–15 (14)	12–15 (14)	14	14–15 (15)	12–16 (15)	14–16 (15)	14–15			15	14–16	13–16 (15)
Pelvic-fin rays	9 (9)	9 (9)	9 (9)	9		9 (9)	9 (9)	9			9	9	9
Dorsal-fin rays	10–12 (11)	10–13 (11)	11–12 (11)	11	11–12 (12)	10–12 (11)	11–12 (11)	11–12			11–12	11–13	11–13 (12)
Anal-fin rays	9–12 (11)	10–12 (10)	10–12 (10)	10	11–12 (11)	9–11 (10)	10–12 (11)	10–11			10	10–12	10–11 (11)
Caudal-fin rays	18–20 (19)	17–22 (19)	18–20 (19)					19			18–19	18–21	
Scales rows above the lateral line	4.5 (4.5)	4.5 (4.5)	4.5 (4.5)	5				4.5					4.5–5.5(4.5)
Scales rows below the lateral line	5.5 (5.5)	5.5 (5.5)	5.5 (5.5)	7				5.5					5.5–7.5(6.5)
Pre-dorsal-fin scales	19–24 (22)	24–27 (25)	19–27 (23)		23–26 (25)	22–27 (25)	26–30 (27)		22–27 (24–25)	26–30 (27–28)	23–25	23–28	20–27 (23)
Lateral-line scales	57–64 (62–63)	64–70 (66–67)	62–67 (63)	63	62–66 (65)	63–65 (65)	68–71 (69)	55–65	62–67 (64–65)	68–71 (69)	61–62	63–70	61–66 (63)
Vertebrae	57–61 (60–61)	62–66 (62–63)	61–63(62)		57–62 (60)	56–59 (58)	61–65 (63)	57–62	56–61 (59)	61–66 (63)	57–59	62–64	57–62 (59)
Measurements (%BL)													
Total length	105.2–119.3 (112.6)	106.7–120.8 (113.1)	110.5–116.9 (114.8)										
Body depth	11.9–17.7 (14.6)	12.4–18.9 (14.5)	13.2–18.4 (14.8)	13.6							12.2–14.5 (13.3)	10.9–16.5 (13.5)	
Head depth	8.8–14.2 (11.9)	9.2–16.5 (11.5)	8.5–14.1 (11.1)	12.1									
Head length	18.0–24.1 (20.9)	17.8–26.8 (20.4)	17.4–21.8 (20.1)	20.7		20.9–23.4 (22.2)	21.6–24.2 (22.9)				22.4–22.6 (22.5)	19.0–24.4 (21.3)	19.4–24.0 (22.3)
Pre- pectoral -fin length	17.2–24.1 (21.3)	16.9–24.3 (21.1)	19.3–22.7 (21.8)			20.5–25.4 (22.9)	21.8–24.7 (23.2)						21.4–36.9 (23.7)
Pre-dorsal-fin length	38.1–45.3 (41.4)	37.9–45.3 (40.9)	36.6–42.5 (40.4)			40.9–44.9 (42.9)	41.0–16.8 (43.9)				41.8	40.0–44.4 (41.5)	39.6–43.5 (41.8)
Pre-pelvic-fin length	31.3–39.4 (34.9)	31.9–39.5 (35.7)	34.2–38.2 (36.3)			34.6–40.9 (37.7)	38.3–40.1 (39.2)				35.3–39.2 (37.2)	19.0–24.4 (21.3)	21.9–41.1 (37.1)
Pre-anal-fin length	70.4–81.4 (74.9)	68.6–78.8 (75.0)	73.7–79.2 (76.6)			72.5–79.6 (76.0)					76.8–86.0 (81.4)	63.9–83.6 (74.9)	71.0–78.0 (74.4)
Pre-adipose-fin length	75.2–83.0 (79.8)	74.3–82.5 (79.5)	71.8–81.7 (79.2)			79.7–85.4 (82.6)					80.0–80.6 (80.3)	68.0–82.9 (78.8)	79.3–83.6 (81.5)
Pectoral-fin length	9.1–13.2 (10.7)	8.6–15.4 (11.9)	7.2–13.0 (11.5)								11.7–12.9 (12.3)	9.6–15.2 (12.2)	9.8–13.5 (11.6)
Measurements (%HL)													
Snout length	17.8–30.2 (23.5)	17.8–29.4 (23.0)	18.3–28.0 (23.4)	28.6							22.6–24.1 (23.3)	22.6–29.6 (27.1)	21.7–26.7 (24.2)
Postorbital length	55.5–82.4 (61.2)	56.0–75.3 (62.5)	56.5–76.9 (63.7)								61.6–62.1 (61.8)	53.5–66.0 (60.4)	58.1–64.2 (61.3)

**Figure 4.**
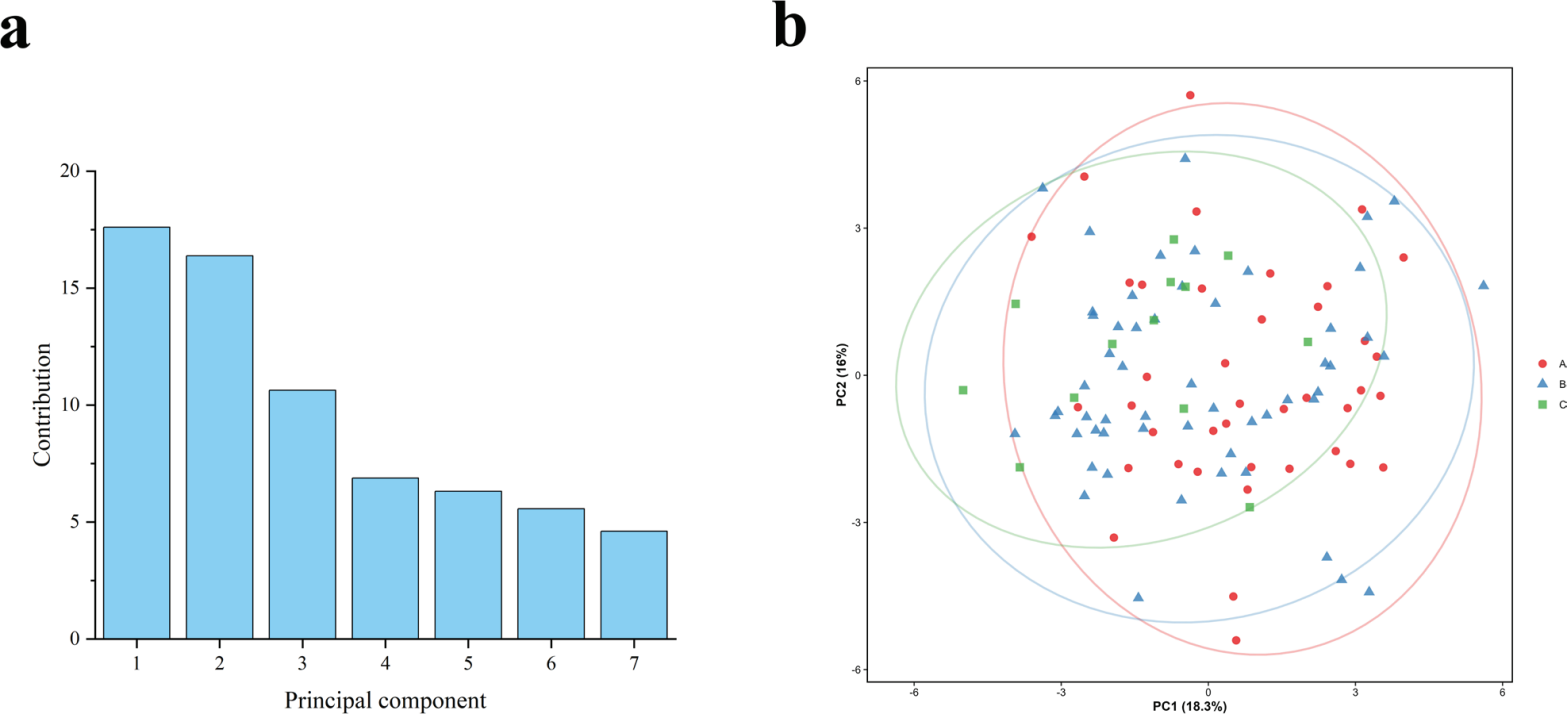
PCA analysis of groups A, B and C. **a**. PCA contribution rates; **b**. PCA plot of specimens from groups A, B, and C based on PC1 and PC2.

One-way ANOVA performed on all morphometric traits showed that none of the indices differed significantly among the three groups (Table 4). Overall, variation in morphometric traits proportions was continuous across the three groups, with no stable interspecific differences or diagnostic taxonomic characters. PCA of all standardized morphological characters showed that the first seven components explained 70.2% of the variance, with PC1 and PC2 accounting for 18.3% and 16.0%, respectively (Fig. 4a). PC1 was mainly related to body depth, head length, and the distance from the dorsal-fin to adipose-fin origin, representing body shape variation. PC2 was associated with snout length and postorbital length, reflecting variation in head proportions. PCA revealed extensive overlap among the three groups on PC1 and PC2, with no distinct clusters (Fig. 4b). Overall, morphometric variation was continuous with no clear group separation, suggesting that the three groups represent variation within a single species. CD among groups ranged from 0.002 to 0.287, all of which were well below the threshold of 1.28 for subspecies recognition.

**Table 4.** One-way ANOVA results for Groups A, B, and C.

Variable	Group		
	A	B	C
WL	1.136 ± 0.027^a^	1.131 ± 0.025^a^	1.148 ± 0.017^a^
BH	0.146 ± 0.013^a^	0.145 ± 0.015^a^	0.148 ± 0.015^a^
HH	0.119 ± 0.014^a^	0.115 ± 0.015^a^	0.113 ± 0.016^a^
HL	0.209 ± 0.014^a^	0.204 ± 0.015^a^	0.202 ± 0.013^a^
SL	0.049 ± 0.005^a^	0.047 ± 0.007^a^	0.047 ± 0.007^a^
EL	0.127 ± 0.010^a^	0.126 ± 0.012^a^	0.128 ± 0.005^a^
PL	0.107 ± 0.009^a^	0.119 ± 0.012^a^	0.115 ± 0.018^a^
AL	0.213 ± 0.016^a^	0.211 ± 0.016^a^	0.218 ± 0.011^a^
CL	0.414 ± 0.014^a^	0.409 ± 0.013^a^	0.404 ± 0.016^a^
GL	0.349 ± 0.018^a^	0.357 ± 0.016^a^	0.363 ± 0.013^a^
FL	0.750 ± 0.023^a^	0.750 ± 0.019^a^	0.766 ± 0.014^a^
DL	0.798 ± 0.018^a^	0.795 ± 0.017^a^	0.792 ± 0.025^a^

Note: The same superscripts mean non-significant difference (*p* >0.05), while different superscripts mean significant difference (*p* <0.05).

### DNA analysis

**Figure 5.**
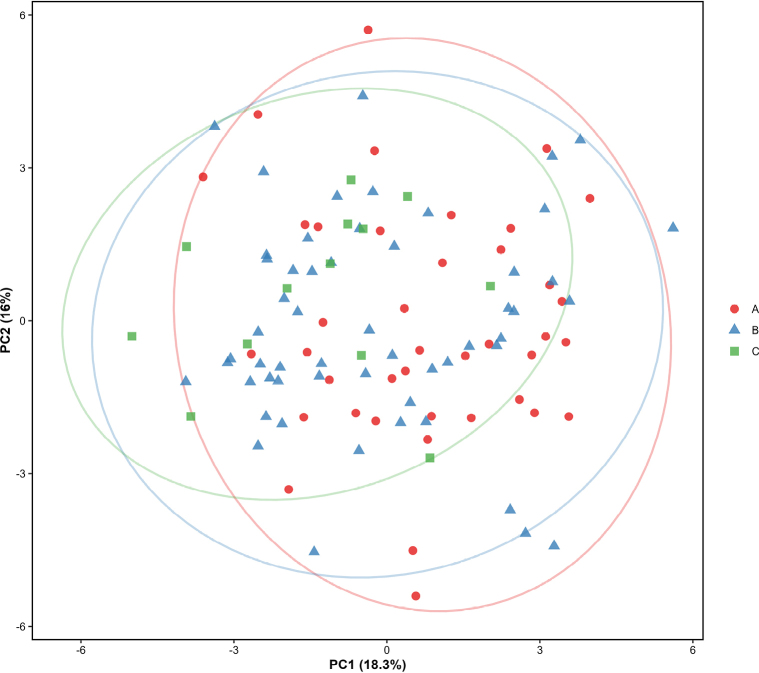
Maximum-likelihood tree of the cytochrome *c* oxidase subunit I gene (619 bp) based on haplotypes of the A–C groups and outgroups (*Saurida macrolepis* and *Saurida fortis*).

One-hundred eight sequences of COI from the three groups were aligned to a length of 619 bp, resulting in 14 haplotypes (GenBank accession number: PX710023–PX710036), five of which were shared (Table 5). Table 6 indicates that the mean genetic distance between Groups A and B was 0.169 ± 0.070%, similar to the intra-group distances of 0.143 ± 0.043% within Group A and 0.180 ± 0.092% within Group B. Pairwise genetic distances among groups did not reach a tenfold difference (Hebert et al. 2003). This indicates that genetic differentiation within and among the groups does not reach the level of interspecific divergence. The maximum-likelihood (ML) phylogenetic tree (Fig. 5) showed that the haplotypes formed a distinct branch relative to the outgroup *Saurida macrolepis* and *S. fortis*. The phylogenetic tree showed no clear differentiation or branching among haplotypes, and no distinct clustering was observed.

**Table 5.** Distribution of haplotypes in the A, B, C groups.

COI	A	B	C	Total
Hap 1	30	32	6	68
Hap 2	3	13	4	20
Hap 3	1	2	1	4
Hap 4	1	1	1	3
Hap 5	1			1
Hap 6	1			1
Hap 7	1			1
Hap 8	1			1
Hap 9	1			1
Hap 10		1	1	2
Hap 11		3		3
Hap 12		1		1
Hap 13		1		1
Hap 14		1		1
Total	40	55	13	108

**Table 6.** Genetic distances within and among the three groups based on COI sequences.

Group	A	B	C
A	0.143 ± 0.043%		
B	0.169 ± 0.070%	0.180 ± 0.092%	
C	0.194 ± 0.084%	0.195 ± 0.099%	0.224 ± 0.116%

## Discussion

Ichthyologists have relied on key traits such as vertebral count, lateral-line scale count, and pre-dorsal-fin scales to classify *Saurida elongata* (=*S. eso*) and *S. microlepis*. Diagnostic thresholds for these traits are largely consistent across studies (Table 7). Both Yamada et al. (2007) and Nakabo (2013) established vertebral counts of 61 and a lateral-line scale count of 65 as diagnostic boundaries. Slight variations are primarily noted in pre-dorsal-fin scale counts. Wu and Zhong (2021) followed Nakabo (2013). Yeo and Kim (2018) proposed more restrictive identification characters, classifying individuals with vertebral counts of 57 and 59 and lateral-line scales of 61 and 62 as *S. elongata* (=*S. eso*), whereas those with vertebral counts of 62–64 and lateral-line scales of 63–70 were identified as *S. microlepis*. The overlap in the diagnostic ranges of vertebral counts (61), lateral-line scales (64), and pre-dorsal-fin scales led to combining of these three traits to group the specimens, as no single trait could reliably differentiate them. When vertebral and lateral-line scales were at their respective boundary values, pre-dorsal-fin scales were additionally considered as a supplementary character. Nevertheless, some specimens could not be clearly assigned to either type based on all three characters. Overall, the specimens were divided into three groups, with Group C comprising specimens exhibiting intermediate traits, indicating that differentiation based on the above key traits is unreliable. Statistical analyses, including one-way ANOVA, PCA, and coefficient of difference analysis, demonstrated that the three groups represent intraspecific variation rather than species-level differentiation.

**Table 7.** Comparison of identification characters for *Saurida* specimens. Species previously recorded as *S. elongata* are essentially *S. eso*.

Characters	Yamada et al. (2007)		Nakabo (2013) and Wu and Zhong (2021)		Yeo and Kim (2018)		This study	
	*S. elongata* (*=S. eso*)	*S. microlepis*	*S. elongata* (*=S. eso*)	*S. microlepis*	*S. elongata* (*=S. eso*)	*S. microlepis*	A	B
Vertebrae	≤61	≥61	56–61 (59)	61–67 (63–64)	57–59	62–64	≤61	≥61
Lateral-line scales	≤65	≥64	59–65	64–70	61–62	63–70	≤64	≥64
Pre-dorsal-fin scales	25–26	26–30 (27–28)	22–27 (25)	26–30 (27)	23–25	23–28	18–24 (23)	24–27 (26)

The taxonomic relationship between *Saurida eso* and *S. microlepis* has undergone multiple modifications, with different scholars proposing divergent points at different stages. Norman (1935) regarded *S. microlepis* as a junior synonym of *S. elongata* (=*S. eso*), and this view was later endorsed by Matsubara and Iwai (1951). Later, Shindo and Yamada (1972) divided *S. elongata* (=*S. eso*) into southern and northern forms, the southern form was characterized by 56–59 (58) vertebrae, 63–65 (65) lateral-line scales, and 22–27 (25) pre-dorsal-fin scales, whereas the northern form possessed 61–65 (63) vertebrae, 68–71 (69) lateral-line scales, and 26–30 (27) pre-dorsal-fin scales. However, their study was based on only five specimens of the southern form. Following this division, Yamada et al. (1986) revised the diagnostic ranges slightly, defining the southern form (Seto Inland Sea and Kii Channel) as having 56–61 (59) vertebrae and 62–67 (64–65) lateral-line scales, and the northern form (Yellow Sea and northern East China Sea) as having 61–66 (63) vertebrae and 68–71 (69) lateral-line scales. Subsequently, Yamada et al. (2007) recognized two distinct species: *S. elongata* (=*S. eso*), vertebrae 61 or less, lateral-line scales 65 or less, and 25–26 pre-dorsal-fin scales; and *S. microlepis*, lateral-line scales 64 or more, vertebrae 61 or more, and 26–30 (27–28) pre-dorsal-fin scales. From a morphological perspective, the *S. elongata* (=*S. eso*) defined by Yamada et al. (2007) resembles the southern form proposed by Yamada et al. (1986), whereas *S. microlepis* corresponds more closely to the northern form. In this study, Group A was distributed south of the Yangtze River estuary, while Group B occurred north of the East China Sea. Groups A and B occurred sympatrically in the northern East China Sea, whereas no sympatric populations were detected in the South China Sea, Taiwan Strait or Yellow Sea. The different distribution patterns of Groups A and B, combined with the divergences in their meristic traits such as vertebral and lateral-line scales, indicate that they may represent distinct populations of the same species *S. eso*. Nevertheless, the observed differentiation is insufficient to support a species-level designation, suggesting that they may represent incipient lineages, and warrant further study.

Russell et al. (2024) re-examined the type specimens of *Saurida elongata* and concluded that it has had a confused nomenclatural history and the name has long been misapplied. As a result, *S. elongata* is redescribed, and *S. eso*Jordan and Herre 1907, previously considered a synonym of *S. elongata*, is resurrected as a valid species. According to the original description and illustrations of Wu and Wang (1931), *S. microlepis* was described with short pectoral fins and a high number of lateral-line scales, features that closely resemble those of *S. eso*, and it has consequently been treated as a synonym of *S. eso*. Therefore, both *S. elongata* and *S. microlepis* in earlier studies correspond to *S. eso*. *Saurida* specimens in this study were consistent with *S. eso*, rather than with the redescribed *S. elongata* of Russell et al. (2024). Russell et al. (2024) reported a mean K2P genetic distance of 0.37 ± 0.17% from 23 COI sequences of *S. elongata* and *S. microlepis* retrieved from Barcode of Life Data System (BOLD) across the Sea of Japan, Korea, Yellow Sea, East China Sea, South China Sea, and the Taiwan Strait. This value was higher than the mean genetic distance between Groups A and B in this study (0.169 ± 0.070%), which may reflect the narrower geographic range of our sampling. Both COI-based genetic distance results support the conclusion of Russell et al., indicating that *S. microlepis* is a junior synonym of *S. eso*. By reassessing the taxonomic status of *S. microlepis* using reproducible, integrative morphological and COI data, this study contributes to *Saurida* systematics and refines diagnostic characters.

In summary, morphological characters showed extensive overlap, with no stable diagnostic traits at the interspecific level. Moreover, genetic analyses revealed that the specimen did not form distinct monophyletic clades, and pairwise genetic distances among groups did not reach a tenfold difference. Based on both morphological and molecular evidence, *Saurida microlepis* Wu & Wang, 1931 should not be regarded as a valid species. Despite this evidence, broader geographic sampling and nuclear gene data are still needed to enable deeper analyses of population genetic diversity and to test whether sympatric populations differ in ecological habits. The study demonstrates the importance of combined morphological and molecular evidence to resolve taxonomic challenges in marine fishes.

## Additional information

### Use of AI

No use of AI was reported.

### Author ORCIDs

Xiaojing Song https://orcid.org/0000-0002-5949-2498

Hanye Zhang https://orcid.org/0000-0002-1645-6796

### Data availability

All of the data that support the findings of this study are available in the main text.
